# Editorial: Coordination of mRNA Transport and Translation With Vesicle and Organelle Trafficking and Dynamics

**DOI:** 10.3389/fcell.2021.800136

**Published:** 2021-11-18

**Authors:** Anni Vedeler, Clive R. Bramham

**Affiliations:** Department of Biomedicine, University of Bergen, Bergen, Norway

**Keywords:** RNP granule, extracellular vesicles, mRNA, endosomes, lysosomes

Mammalian cells have complex endomembrane systems and vesicular and tubular transport routes that connect the cellular internal compartments and establish the biosynthetic and endocytic pathways. Originally, the sorting and transport activities of these pathways, by ensuring the asymmetric distribution of endoplasmic reticulum (ER)-derived proteins and lipids, were thought to provide the cells with the ability to build distinct functional domains. However, studies carried out during the last decades have revealed that functional polarity of cells can be determined already at the post-transcriptional level *via* the sorting and transport of mRNAs (Martin and Ephrussi, 2009). Thus, specific sets of mRNAs together with RNA-binding proteins in dynamic ribonucleoprotein complexes, called RNP granules, can move in a motor-dependent manner along cytoskeletal tracks from the cell center to peripheral locations docked onto moving vesicles (Kharod et al.). Classical examples of these processes include long-range microtubule-based transport of selected mRNAs *via* membraneless RNP granules, allowing fast synaptic re-modeling (Bramham and Wells, 2007; Holt et al., 2019; Dalla Costa et al., 2021). As summarized in this Research Topic volume, recent work now reveals coordinate delivery of RNP granules with membranous organelles such as endosomes and lysosomes to neuronal axons and dendrites, as well as transport of mitochondrial mRNAs inside endosomes (Kharod et al.; Rajgor et al.). The formation of RNP granules occurs *via* liquid-liquid phase separation (Rajgor et al.). Namely, RNP granules lack a membrane and thus phase separation occurs *via* highly dynamic RNA—protein interactions. The coiled-coil structure is significantly overrepresented in RNA-binding proteins, as found by prediction using databases combined with different algorithms (softwares), and often localized to liquid-liquid phase separated neuronal granules (Ford and Fioriti). The quest to visualize the regulation and function of RNP granules *in situ* has been enormously facilitated by developments in optics and genetically-encoded probes, allowing detection of nascent mRNA and protein at the single molecule level (Kharod et al.; Ford and Fioriti). The RNA-binding motifs and the coiled-coil structure found in RNA-binding proteins are in proximity but never overlapping (Ford and Fioriti) suggesting that the coil-coiled motifs are involved in interaction with protein ligands. Interestingly, it has been proposed that coiled-coil oligomerization under certain circumstances may lead to β-sheet formation, which in turn may lead to the formation of insoluble aggregates and various neurodegenerative diseases (Ford and Fioriti).

Interestingly, it was shown that a subset of RNP granules containing mRNAs necessary for growth cone morphology, tether onto lysosomes *via* Annexin A11, and the two organelles are transported together in cortical neurons. Amyotrophic lateral sclerosis (ALS)-associated mutations in Annexin A11 disrupted this coordinated transport and contribute to the development of ALS (Liao et al., 2019) (Kharod et al.; Rajgor et al.; Ford and Fioriti). The annexin family may turn out to be an interesting protein family in the coordination and regulation of vesicle and mRNA transport/translation as at least one member of the family of Ca^2+^-dependent lipid-binding proteins, Annexin A2, is involved in the localization (Mickleburgh et al., 2005) of c-*myc* mRNA and binds to the internal ribosomal entry sites (IRESs) of c-*myc* (Strand et al., 2021) and p53 (Sharathchandra et al., 2012) mRNAs.

Once transported to the synapse, translation of the mRNAs occurs by an activity-dependent mechanism and is fine-tuned by miRNAs and fueled by nearby mitochondria (Kharod et al.; Rajgor et al.). Post-translational modifications of the proteins in the RNP granules play a key role in releasing the mRNAs from the granules to be actively translated at the synapse (Rajgor et al.). Thus, local protein synthesis allows the cells to rapidly respond to external signals on-site.

The co-ordination of mRNA transport with endosomal/lysosomal transport is important for intracellular communication while transport of lipids, proteins, and RNA in extracellular vesicles (EVs) is important for intercellular communication (Prieto-Vila et al.). EVs consist of microvesicles released by budding from the plasma membrane, apoptotic bodies, and exosomes derived from the endocytic pathway by release of intraluminal vesicles from multivesicular bodies at the plasma membrane. Diseases, including cancer, appear to increase the amount of released/shed EVs. The mRNA cargo and other types of RNA in EVs can modulate physiological responses in the recipient cells. It has been shown that the mRNAs are translated, and the protein product exerts functional effects in recipient cells (Prieto-Vila et al.). Specific elements in both mRNA and miRNA control the loading of these RNAs into EVs, thus specifically targeting them for intercellular communication. In addition to the ESCRT protein machinery, specific proteins such as activity-regulated cytoskeleton associated protein (Arc) in neurons and LC3/ATG8 in the autophagy pathways, participate in loading of RNA into EVs. Arc self-assembles into virus-like capsids which harbor Arc mRNA inside (Ashley et al., 2018; Pastuzyn et al., 2018). The assembly of capsids is also promoted by Arc protein interaction with RNA, mediated by a coiled-coil motif (Eriksen et al., 2021). Tumor-derived EVs appear to play a role in all stages of cell transformation, such as metastasis, neo-angiogenesis, and drug resistance. Furthermore, they prime the tumor microenvironment to become more favorable for the tumor (Prieto-Vila et al.). EVs also appear to be involved in immunosuppression. Specific mRNAs from tumor-derived EVs can be used as biomarkers for cancer diagnosis and for treatment prognosis. Thus, inhibition of EVs has been considered as a therapeutic approach but this is still a method to be developed further (Prieto-Vila et al.).

Thus, it has become evident that membrane traffic and mRNA transport in mammalian cells are coordinated processes, which not only share common pathways and regulatory factors, but also respond to the same extracellular signals. These closely coordinated processes ensure specificity in terms of the mRNAs involved and are highly energy efficient, which becomes particularly evident in the gigantic neurons. Also, the communication of cells with their environment is tightly regulated and can be modulated by the protein and RNA cargoes enclosed in the EVs. As pointed out by the papers of this Frontiers Research Topic, several outstanding questions remain to be addressed by future work. Thus, the era of the present topic: “Coordination of mRNA transport and translation with vesicle and organelle trafficking and dynamics” is only at its dawn and much effort is needed to reveal the post-translational modifications involved the joint regulation of these processes, as well as to identify the specific proteins acting at the crossroads of the coordinated transport events.
